# Association of adherence to the EAT-Lancet diet and risk of uterine cancer: a prospective cohort from the UK Biobank

**DOI:** 10.3389/fnut.2026.1801964

**Published:** 2026-06-17

**Authors:** Lu Liu, Lu Ding, Zhen Chen, Lin Tang, Tianyuan Su, Nan Yang, Qi Yan, Zuoling Yang, Zhiyi Wei, Rong Li, Ruxianguli Aimuzi

**Affiliations:** State Key Laboratory of Pathogenesis, Prevention and Treatment of High Incidence Diseases in Central Asia, College of Public Health, Xinjiang Medical University, Urumqi, China

**Keywords:** cancer prevention, dietary pattern, EAT-Lancet diet, UK Biobank, uterine cancer

## Abstract

**Background:**

The EAT-Lancet dietary pattern is a healthy eating model linked to multiple health benefits. However, whether it is associated with uterine cancer, including malignancies of the isthmus uteri, endometrium, myometrium, fundus uteri, overlapping lesions of the corpus uteri, and uterus of unspecified site, remains unexplored.

**Methods:**

This study comprised 114,572 women without uterine cancer at baseline. Participants who had at least one 24-h dietary recall record in the UK Biobank were included. Cox proportional hazards models were applied to estimate hazard ratios (HRs) and 95% confidence intervals (95% CIs) for incident uterine cancer across the EAT-Lancet diet index. Cox proportional hazards models were adjusted for age, ethnicity, Townsend Deprivation Index, total energy intake, smoking status, alcohol intake, and physical activity.

**Results:**

This study had an average follow-up period of 13.26 years. During this time, 694 new cases of uterine cancer were recorded. Participants with higher adherence to the EAT-Lancet diet (index ≥ 12) had a lower risk of uterine cancer than those in the lowest adherence group (HR = 0.706, 95% CI: 0.547–0.911). Each 1-point increase in the EAT-Lancet diet index was associated with 6.9% lower risk of uterine cancer (HR = 0.931, 95% CI: 0.874–0.992).

**Conclusion:**

Higher adherence to the EAT-Lancet dietary pattern was associated with a lower risk of uterine cancer.

## Graphical abstract

**Figure:**
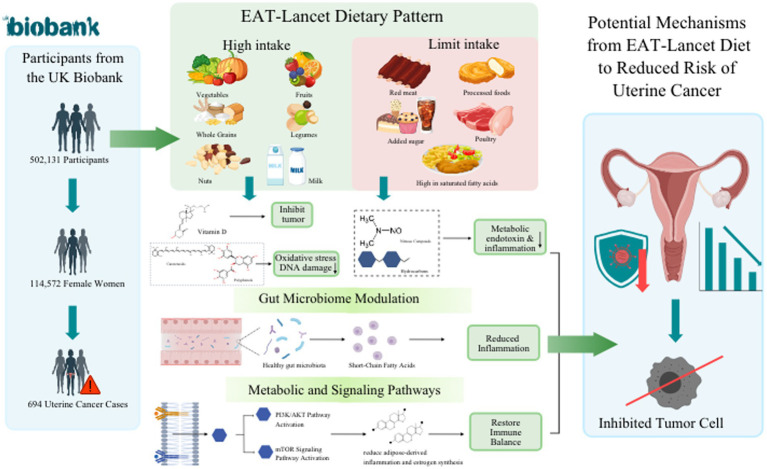
Created with MedPeer (medpeer.cn).

## 1 Introduction

Cancer has become a critical public health concern facing humanity in recent years (1, 2). Within gynecologic oncology, uterine cancer (including endometrial cancer and other malignant tumors of the uterine body) has been rising globally, and warrants increased attention (3, 4). The International Agency for Research on Cancer reports that approximately 20 million cancers were diagnosed worldwide in 2022, with uterine cancer accounting for about 420,000 of these cases (5). Uterine cancer is a malignant tumor with a relatively high incidence rate among women. Contributions are disproportionate to the global burden of cancer, with higher incidence in high-income settings and higher mortality in low and middle-income regions (6). Because advanced-stage disease and high-risk pathological subtypes are associated with poor prognosis, identifying modifiable risk of factors is essential for prevention (7). Type 2 diabetes and obesity are well-established risk factors for uterine cancer (8), and physical inactivity, smoking, and alcohol consumption have also been associated with increased risk (9–11). Collectively, these observations highlight the central role of metabolic and lifestyle factors in uterine cancer etiology. However, the potential contribution of specific dietary patterns to uterine cancer risk remains underexplored, despite diet being a major modifiable lifestyle factor. Investigating the association between dietary patterns and uterine cancer may therefore provide important insights for primary prevention strategies.

Studies have reported that greater consumption of fruits and vegetables reduces the probability of uterine fibroid development, whereas high intake of red meat promotes fibroid growth (12). Low dietary intake of fiber, vitamin C, and vitamin D is associated with a high risk of cervical cancer and uterine fibroids. In contrast, consuming antioxidants-rich diets, including vitamin A, vitamin E, and carotenoids, may suppress the development and growth of cervical cancer (13, 14). However, single nutrients or foods cannot fully capture the complexity of overall dietary exposure. Dietary pattern analysis offers a more comprehensive approach to examining diet-disease associations (15). Emerging evidence suggests that healthy dietary patterns, such as the Mediterranean diet and DASH diet, are associated with reduced risk of various chronic diseases, including certain cancers (16–18). As can be seen in previous research, proinflammatory dietary patterns are positively correlated with breast cancer (19). In 2019, the EAT-Lancet Commission introduced the EAT-Lancet dietary pattern (20), which emphasizes human health and environmental sustainability (21). Compared with the Mediterranean diet, this dietary pattern prioritizes plant-based foods while restricting red and processed meats. It encourages moderate consumption of dairy products and fish while restricting food high in added sugars and saturated fats. In particular, this dietary pattern encourages greater use of legumes and whole grains (22). Evidence from previous studies indicates a positive association between following the EAT-Lancet dietary pattern and incidence of stroke, fatty liver disease, anxiety disorders, and depression (23–27). Still, its association with uterine cancer is largely unexplored. Therefore, using a large prospective cohort from the UK Biobank, this study investigated the association between adherence to this dietary pattern and uterine cancer risk.

## 2 Methods

### 2.1 Study participants

This analysis was based on the UK Biobank, which enrolled 502,131 adults aged 37 to 73 between 2006 and 2010 (28). All participants attended an assessment visit at 22 centers across England, Wales, and Scotland. During these visits, participants completed a self-administered touchscreen questionnaire on sociodemographic characteristics, lifestyle factors and dietary habits. Additionally, standardized anthropometric measurements and biospecimen collection (29). All participants provided electronically signed informed consent. The UK Biobank was ethically approved by the National Health Service (NHS) North West Research Ethics Committee. Additional details of the touchscreen questionnaire and study resources are available from the UK Biobank website (www.ukbiobank.ac.uk/resources/).

**Figure 1:**
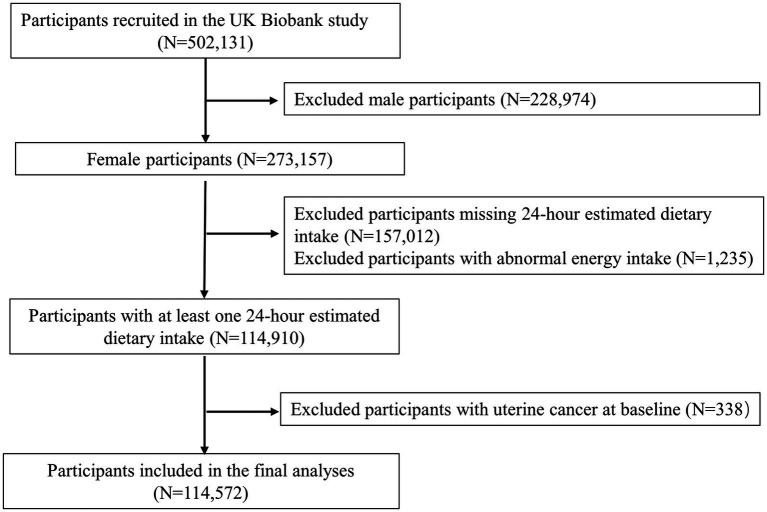
Study subject screening flowchart.

A total of 387,559 participants were excluded from the study cohort, including 228,974 male participants, 158,247 individuals with incomplete dietary data or abnormal energy intake (< 500 or > 4,000 kcal/day in women), and 338 participants with uterine cancer at baseline. Finally, 114,572 participants were included in the analysis (Figure 1).

### 2.2 EAT-Lancet diet index

Dietary intake was evaluated with the Oxford WebQ, an online 24-h dietary recall questionnaire, which was used in the UK Biobank to collect information on intake of 206 individual foods and 32 individual beverages during the previous 24 h (30). A total of five 24-h dietary recalls were administered, with the initial one conducted at the assessment center between April 2009 and September 2010. Online data collection occurred from April 2009 to September 2010, with online cycles conducted from February to April, June to September, and October to December 2011 and April to June 2012. The questionnaire’s validity has been demonstrated using participants’ 24-h dietary recall (31), biomarkers (32), and test–retest reproducibility (33). Nutritional intakes were calculated with reference to the UK Biobank food composition tables. Food and beverage consumption was estimated from reported portion sizes for each item (34). The number of individuals completing at least one 24-h dietary recall within the study period (April 2009 to June 2012). This diet index was derived from the average across all available recalls per participant, using the portion sizes and scoring criteria defined for this study (Supplementary Table S1), to reflect adherence to the EAT-Lancet dietary pattern. Additional details on dietary intake processing and index construction are provided in Supplementary material (Methods 2).

Each dietary component was scored based on compliance with the EAT-Lancet dietary criteria (Supplementary Table S2). The total EAT-Lancet diet index was calculated as the sum of all component scores, with equal weighting for each component. The index ranged from 0 to 14, with higher scores indicating greater adherence. For analysis, the index was categorized into four groups: ≤9, 10, 11, and ≥12. The scoring methodology primarily followed that of Knuppel et al. (24). Proportion of subjects meeting the Knuppel EAT-Lancet diet index recommendations (Supplementary Table S3).

### 2.3 Incident uterine cancer

We defined uterine cancer by ICD-10 codes C54 and C55. Incident uterine cancer was identified through cancer registries in England, Scotland, and Wales (Supplementary Table S4). We extracted information on uterine cancer events and deaths from hospital records, cancer registries, and death registries electronically. Women were followed from the date of attendance at the assessment center until the date of first uterine cancer diagnosis, death, or the end of follow-up (England: 31 October 2022, Wales:31 August 2022, Scotland: 31 May 2022).

### 2.4 Statistical analysis

We assessed the normality of all continuous variables in the 114,572 participants with the Kolmogorov–Smirnov test and visualized their distributions using histograms. Variables with a normal distribution were presented as mean (standard deviations, SD), whereas median (interquartile range, IQR) was used to describe baseline and other non-normally distributed variables. Categorical variables were described as numbers (percentage). Participants who lacked data or answered “prefer not to answer” or “do not know” for these items were grouped into a “Missing” category for the corresponding categorical variables. A summary of the amount of missing data was shown in Supplementary Table S5.

Follow-up time (person-years) was calculated from the date of the first 24-h dietary recall to the earliest of uterine cancer diagnosis, death, or study end. We used multivariable Cox proportional hazards models to estimate hazard ratios (HRs) and 95% confidence intervals (CIs) for the association between the EAT-Lancet diet index and uterine cancer risk. The lowest category (≤9 points) served as the reference group. We also examined the association per 1-point increase in the diet index. Trend tests were performed by modeling the median value of each category as a continuous variable. The proportional hazards assumption was assessed using Schoenfeld residuals, and no evidence of violation was observed.

A set of covariates including age, sex, ethnicity, the Townsend Deprivation Index (TDI), total energy intake, smoking status, alcohol consumption, and physical activity, was selected based on previous studies (35). Total energy intake was measured with one 24-h dietary recall questionnaire, whereas all other covariates were extracted from baseline data. We fitted three Cox proportional hazards models with progressively increasing adjustment. Model 0 was an unadjusted (crude) model. Model 1 adjusted for age, TDI, ethnicity, and total energy intake to account for baseline demographic and socio-economic differences as well as overall dietary quantity. Model 2 was further adjusted for lifestyle factors, including smoking status, alcohol consumption, and physical activity, to reduce residual confounding by health behaviors correlated with diet. Detailed methodology for covariate assessment was provided in Supplementary material (Methods 1), and specific covariate definitions and coding were summarized in Supplementary Table S6.

We used the Cox proportional hazards model to assess the association between the components of the Knuppel EAT-Lancet index and the risk of uterine cancer (Supplementary Table S7). We further conducted subgroup analyses of covariates to assess the association between the EAT-Lancet Diet Index and the risk of uterine cancer, evaluated potential effect modification using interaction tests (Supplementary Table S8).

Given that BMI and waist circumference might be involved in how the EAT-Lancet diet influences disease progression, we also performed the mediation analysis to explore whether either BMI or waist circumference mediates this association in uterine cancer. We estimated the direct effect (DE), the effect of exposure on outcomes that is not mediated by the mediator, and the indirect effect (IE), the effect of exposure on outcomes that is mediated by the mediator. The bootstrap method (with 1,000 repetitions) was employed to estimate the mediation effect and its 95% CI, with direct, indirect, and mediation proportions calculated separately (Supplementary Table S9).

Several sensitivity analyses were performed to assess the robustness of our findings (Supplementary Table S10). First, participants with only one 24-h dietary recall, assessed using the Oxford WebQ, were excluded. Second, the follow-up period was recalculated starting from the most recent dietary assessment. Third, we excluded participants with missing baseline covariate data to evaluate the reliability of data imputation. Finally, to limit the possibility of confounding by reverse causality, uterine cancer cases diagnosed within the first five years after follow-up were excluded. All analyses were performed using *R* 4.4.2, and statistical significance was determined by a two-tailed *p* < 0.05.

## 3 Results

### 3.1 Baseline characteristics

A total of 114,572 participants were included in this study. Mean age (SD) was 55.65 (7.83) years. During a follow-up of 13.26 (IQR = 12.68 to 14.04) years, 694 incident uterine cancers were identified. The EAT-Lancet diet index ranged from 5 to 14 points, with a median (IQR) of 10 (10, 11). Individuals with a higher index tended to be older, were more often nonsmokers, engaged in regular physical activity, and had lower BMI, waist circumference, and energy intake (Table 1).

**Table 1:** Comparison of baseline characteristics across the Knuppel EAT-Lancet index categories (range, 0–14 points) (*N* = 114,572).

Participant characteristics	Total (*N* = 114,572)	the EAT-Lancet index			
		≤9 (*N* = 24,237)	10 (*N* = 34,380)	11 (*N* = 34,547)	≥12 (*N* = 21,408)
Age (y), mean (SD)	55.65 (7.83)	54.52 (7.99)	55.61 (7.87)	56.12 (7.74)	56.23 (7.62)
Ethnic, *N* (%)					
White	109,377 (95.47)	23,108 (95.34)	32,905 (95.71)	33,010 (95.55)	20,354 (95.08)
Other	4,910 (4.29)	1,071 (4.42)	1,383 (4.02)	1,452 (4.20)	1,004 (4.69)
Missing	285 (0.24)	58 (0.24)	92 (0.07)	85 (0.23)	50 (0.23)
TDI, *N* (%)					
Above median, (≥ − 2.28)	57,186 (49.91)	12,512 (51.62)	16,824 (48.94)	16,980 (49.15)	10,870 (50.78)
Below median, (<−2.28)	57,091 (49.83)	11,656 (48.09)	17,460 (50.79)	17,491 (50.63)	10,484 (48.97)
Missing	295 (0.26)	69 (0.28)	96 (0.28)	76 (0.22)	54 (0.25)
Smoking status, *N* (%)					
Never	69,434 (60.60)	14,278 (58.91)	20,873 (60.71)	21,313 (61.69)	12,970 (60.58)
Previous	37,216 (32.48)	7,743 (31.95)	11,061 (32.17)	11,143 (32.25)	7,269 (33.95)
Current	7,633 (6.66)	2,159 (8.91)	2,357 (6.86)	1996 (5.78)	1,121 (5.24)
Missing	289 (0.26)	57 (0.23)	89 (0.26)	95 (0.28)	48 (0.23)
Alcohol status, *N* (%)					
Never	4,781 (4.17)	1,002 (4.13)	1,414 (4.11)	1,488 (4.31)	877 (4.10)
Previous	3,556 (3.10)	736 (3.04)	1,052 (3.06)	1,053 (3.05)	715 (3.34)
Current	106,119 (92.62)	22,474 (92.73)	31,880 (92.73)	31,972 (92.55)	19,793 (92.46)
Missing	116 (0.10)	25 (0.10)	34 (0.10)	34 (0.09)	23 (0.10)
Having regular physical activity, *N* (%)					
Yes	62,702 (54.73)	12,038 (49.67)	18,253 (53.09)	19,590 (56.71)	12,821 (59.89)
No	46,972 (41.00)	11,149 (46.00)	14,613 (42.50)	13,494 (39.06)	7,716 (36.04)
Missing	4,898 (4.28)	1,050 (4.33)	1,514 (4.40)	1,463 (4.23)	871 (4.07)
BMI (kg/m^2^), mean (SD)	26.53 (5.00)	27.35 (5.35)	26.78 (5.04)	26.29 (4.86)	25.58 (4.56)
Waist circumference (cm), mean (SD)	83.37 (12.15)	85.35 (12.74)	83.99 (12.22)	82.79 (11.87)	81.09 (11.35)
Total energy intake (kcal/day), mean (SD)	1899.68 (479.99)	1985.37 (487.14)	1929.23 (476.34)	1883.00 (468.04)	1782.12 (471.71)
Dietary component (g/day), median (Q1, Q3)					
Whole grains	130.00 (65.00, 162.50)	130.00 (43.30, 130.00)	130.00 (65.00, 162.50)	160.00 (65.00, 162.50)	130.00 (65.00, 173.30)
Potatoes	38.70 (0.00, 58.00)	38.70 (0.00, 58.00)	38.70 (0.00, 58.00)	38.70 (0.00, 58.00)	29.00 (0.00, 58.00)
Vegetables	250.00 (133.30, 400.00)	150.00 (83.30, 283.30)	220.00 (100.00, 366.70)	293.80 (200.00, 425.00)	337.50 (241.30, 500.00)
Fruits	251.40 (150.00, 396.00)	120.00 (24.00, 293.30)	237.00 (136.50, 372.00)	300.00 (174.00, 424.50)	324.00 (222.00, 474.00)
Dairy food	180.00 (64.50, 270.00)	149.40 (43.00, 258.00)	172.00 (64.50, 258.00)	180.00 (71.50, 270.00)	180.00 (90.00, 306.00)
Red meat	40.00 (0.00, 80.00)	80.00 (40.00, 100.00)	60.00 (0.00, 80.00)	40.00 (0.00, 80.00)	0.00 (0.00, 20.00)
Poultry	0.00 (0.00, 60.00)	0.00 (0.00, 80.00)	0.00 (0.00, 80.00)	0.00 (0.00, 40.00)	0.00 (0.00, 26.70)
Eggs	0.00 (0.00, 30.00)	30.00 (0.00, 60.00)	0.00 (0.00, 30.00)	0.00 (0.00, 15.00)	0.00 (0.00, 0.00)
Fish	0.00 (0.00, 50.00)	0.00 (0.00, 33.30)	0.00 (0.00, 50.00)	0.00 (0.00, 50.00)	0.00 (0.00, 75.00)
Legumes	0.00 (0.00, 75.00)	0.00 (0.00, 66.70)	0.00 (0.00, 66.70)	0.00 (0.00, 75.00)	0.00 (0.00, 75.00)
Nuts	0.00 (0.00, 0.00)	0.00 (0.00, 0.00)	0.00 (0.00, 0.00)	0.00 (0.00, 0.00)	0.00 (0.00, 14.00)
Added fats	1.52 (1.24, 1.88)	1.45 (1.19, 1.75)	1.47 (1.20, 1.79)	1.52 (1.24, 1.880)	1.73 (1.37, 2.19)
Added sugar	102.00 (41.30, 207.80)	137.00 (72.00, 256.00)	120.00 (60.00, 231.50)	97.00 (39.30, 198.50)	30.00 (9.00, 119.85)

Data were summarized as frequency (%), mean (SD) or median (IQR) according to the data type.

### 3.2 Relationship between following the EAT-Lancet diet and uterine cancer risk

**Figure 2:**
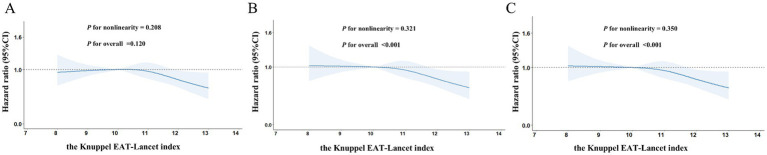
The dose–response associations between the Knuppel EAT-Lancet index and risks of incident uterine cancer. The Knuppel EAT-Lancet index was categorized into four groups (≤9, 10, 11, ≥12 points), with higher scores indicating greater adherence to the EAT-Lancet dietary pattern. Unadjusted **(A)**, with adjusted for age, TDI, ethnicity, and energy intake **(B)**, with adjusted for age, TDI, ethnicity, energy intake, smoking status, alcohol intake and physical activity **(C)**. Hazard ratios (HRs) and 95% confidence intervals (CIs) were estimated using Cox proportional hazards models incorporating a restricted cubic spline for the Knuppel EAT-Lancet index. The knots for the restricted cubic spline were positioned at the 5th, 35th, 65th, and 95th percentiles of the Knuppel EAT-Lancet index. *p* values for non-linearity and for the overall association were derived from Wald tests.

The restricted cubic spline analysis (Figure 2) indicated a linear dose–response for the EAT-Lancet index in relation to uterine cancer, with no evidence of deviation from linearity in any model (all *p* values for nonlinearity > 0.05). In model 0 (unadjusted), women in the highest score category (EAT-Lancet index ≥ 12) had a significantly reduced risk compared with those in the lowest category (Table 2), with a hazard ratio of 0.756 (95% CI: 0.588–0.973). After further adjusting for covariates in Models 1 and 2, this association remained statistically significant (Model 1: HR = 0.703, 95% CI: 0.545–0.906; Model 2: HR = 0.706, 95% CI: 0.547–0.911).

**Table 2:** Relationship between the Knuppel EAT-Lancet index (0–14 points) and uterine cancer risk (*N* = 114,572).

Knuppel EAT-Lancet index	N_case_/N_total_	Model 0^a^		Model 1^b^		Model 2^c^	
		HR (95% CI)	*p* value	HR (95% CI)	*p* value	HR (95% CI)	*p* value
≤9	151/24237	REF		REF		REF	
10	219/34380	1.024 (0.832–1.260)	0.822	0.978 (0.795–1.204)	0.835	0.976 (0.793–1.202)	0.820
11	223/34547	1.037 (0.844–1.275)	0.730	0.968 (0.787–1.192)	0.760	0.968 (0.786–1.192)	0.758
≥12	101/21408	0.756 (0.588–0.973)	0.030	0.703 (0.545–0.906)	0.007	0.706 (0.547–0.911)	0.007
*P* for trend		0.074		0.016		0.017	
1-point increment in diet score	694/114572	0.951 (0.895–1.011)	0.110	0.930 (0.873–0.990)	0.023	0.931 (0.874–0.992)	0.026

^a^Model 0: Unadjusted. ^b^Model 1: With adjusted for age, TDI, ethnicity, and energy intake. ^c^Model 2: With adjusted for age, TDI, ethnicity, energy intake, smoking status, alcohol intake and physical activity.

### 3.3 Subgroup analyses

There was a statistically significant overall association between the EAT-Lancet diet index and uterine cancer (Supplementary Table S8). This findings was consistent across age, TDI, smoking status, alcohol consumption, physical activity, and BMI. Specifically, a higher diet index was linked to lower uterine cancer risk among participants with below-median TDI (HR = 0.649, 95% CI: 0.441–0.956), never smokers (HR = 0.686, 95% CI: 0.495–0.951), and those who engaged in regular physical activity (HR = 0.682, 95% CI: 0.488–0.953). However, the interaction analysis showed no statistically significant interaction between the EAT-Lancet diet index and any stratification factor (all interaction *p*-values were ≥0.05).

### 3.4 Mediating effects analyses

The mediated analyses indicated that BMI accounted for 49.4% of the association between the EAT-Lancet diet index and uterine cancer risk (95% CI: 0.249–1.908; *p* = 0.014). Similarly, 74.3% (95% CI: 0.243–1.905; *p* = 0.006) of the association was mediated by waist circumference (Supplementary Table S9).

### 3.5 Sensitivity analyses

In sensitivity analyses, the inverse association between the EAT-Lancet diet index and uterine cancer risk remained consistent when using the latest dietary survey for follow-up, excluding participants with missing covariates, excluding cases diagnosed within the first five years, excluding participants with a family history of cancer, and excluding those who used oral contraceptives (Supplementary Tables S10, S11). Kaplan–Meier curves showed consistently higher event-free survival in the high diet index group (≥ 12 points) compared with the low group (< 12 points) across all models (Supplementary Figure S1).

## 4 Discussion

In this large prospective cohort study of over 114,000 women, we found that greater adherence to the EAT-Lancet dietary pattern was associated with a significantly lower risk of uterine cancer. This association was particularly strong among women with higher TDI scores (indicating higher socioeconomic status), never-smokers, and those who were physically active. Importantly, adiposity measures, including BMI and waist circumference, substantially mediated this association, suggesting that body weight management may be a key pathway through which dietary patterns influence uterine cancer risk.

Multiple studies report associations between following the EAT-Lancet pattern and various health outcomes. These include disease of cardiovascular disease (36), dementia (37, 38), rheumatoid arthritis (39), liver disease (23), colorectal cancer (40), lung cancer (41), kidney cancer (42), and all-cause mortality (43). Numerous studies confirmed that following this dietary pattern is associated with each of these respective outcomes. Within the existing literature, this study is the first to comprehensively examine how adherence to this dietary pattern relates to uterine cancer risk in the UK Biobank. These findings have important clinical and public health implications. The EAT-Lancet dietary pattern is characterized by high intakes of whole grains, vegetables, fruits, and legumes, and low intakes of red meat, processed meat, added fats, and sugars. Compared to focusing on individual foods or nutrients, the EAT-Lancet index provides a more comprehensive reflection of a holistic, food-based dietary pattern. Our findings suggest that stricter adherence to this dietary pattern is associated with a significant reduction in the risk of endometrial cancer. At the population level, improving this modifiable dietary pattern is expected to significantly reduce the disease burden, particularly when combined with other preventive strategies such as weight management and regular physical activity. However, given the observational nature of our study, these findings should be interpreted as associations rather than proof of a causal effect of the EAT-Lancet diet on uterine cancer risk.

In addition, we found that participants with a lower TDI (< median). An even stronger inverse association was evident between this diet index and uterine cancer risk. This pattern may be related to the fact that individuals with low TDI often have higher educational levels, greater economic status, and stronger health awareness, and better access to nutritious foods, thus being more likely to adopt and adhere to the EAT-Lancet dietary pattern (44). Similarly, comparable results were also found among never smokers and individuals engaging in regular physical activity. Smoking and unhealthy diet behaviors often cluster together (45, 46), whereas regular exercise and a healthy diet would promote each other, enhance the overall diet structure, and increase the likelihood of forming the EAT-Lancet dietary pattern (47). It should be acknowledged that the above-mentioned subgroups’ differences may be partially explained by residual confounding or reporting bias rather than true effect modification; this conclusion was not supported by statistically significant interactions in the stratified analysis.

Mediation analysis revealed that obesity plays an important mediating role in linking adherence to this dietary pattern with uterine cancer risk, whereas the mediating role of BMI in this association remains unclear. Abdominal obesity differs from BMI, which represents overall body size. Waist circumference is a better indicator of visceral fat mass and metabolic abnormalities (48). Visceral fat is metabolically active tissue that has been linked to the development of insulin resistance, a persistent low-grade inflammatory state, and hormonal disturbances. All of these can promote carcinogenesis (48–50). Thus, the observed association between adherence to this dietary pattern and uterine cancer risk may be partially explained by a reduction in abdominal obesity. In addition, the EAT-Lancet dietary pattern may have other beneficial effects on uterine cancer by improving metabolic and hormonal balance and reducing inflammation, independent of body weight (51, 52). Although the mediated proportion estimates are imprecise and should be interpreted with caution, the overall evidence supports targeting both dietary optimization and central obesity management as complementary strategies to reduce the risk of uterine cancer.

Although the causal mechanisms linking this diet index to uterine cancer are not yet fully understood, several plausible biological pathways may account for the observed protective association. To begin with, the EAT-Lancet diet contains large amounts of plant compounds, fiber, vitamins, and minerals (53). These components are found in fruits and vegetables, and these foods affect bacteria in the digestive system, producing compounds with short-chain fatty acids (12, 54–56). These compounds exhibit anti-inflammatory and anticancer effects. Vitamin D demonstrates effects that inhibit tumor development, and this vitamin reduces cell growth in Uterine Fibroids and decreases their size in studies using animals and in studies involving treatment of individuals (57, 58). Antioxidants such as carotenoids and polyphenols may further mitigate oxidative stress and DNA damage (59, 60). The diet shows a pattern of decreased use of red and processed meat, which reduces exposure to nitroso compounds and hydrocarbons with multiple rings (61). These compounds relate to an increased risk of cancer. Third, improved insulin sensitivity and lipid metabolism may attenuate PI3K/AKT activation, reduce adipose-derived inflammation and estrogen synthesis, and help restore immune balance (62–64). Fourth, a growing body of evidence indicates that the mTOR pathway acts as a central hub integrating nutrient sensing, hormonal signaling, and metabolic reprogramming in the development of endometrial cancer. Adherence to the EAT-Lancet dietary pattern, characterized by lower total energy intake and reduced consumption of refined carbohydrates and saturated fats, may suppress excessive mTOR activation, help preserve mitochondrial function, and attenuate chronic inflammation, thereby potentially lowering endometrial cancer risk (65–67). Additionally, restricting sugars and saturated fats may decrease metabolic endotoxin load and low-grade inflammation. Overall, these findings imply that greater adherence to the EAT Lancet diet may lower uterine cancer risk through its integrated effects on inflammation, metabolism, and hormonal regulation (68–73).

There are multiple strengths in our study. First, the UK Biobank represents a large study following individuals over time across multiple locations. This approach provides data from a substantial sample with extended follow-up, and the analysis shows sufficient power to examine uterine cancer outcomes. Second, as far as we are aware, few investigations have considered dietary patterns as determinants of uterine cancer, and this study helps to fill this research gap. Finally, the studies have also examined the EAT Lancet dietary pattern in relation to uterine cancer, which provides evidence for prevention approaches.

This study also has several limitations. First, dietary intake was assessed using repeated 24-h dietary recalls rather than prospective records of usual diet. In the UK Biobank, up to five 24-h dietary recalls were administered; however, each 24-h recall still captures only short-term intake and is subject to recall errors and day-to-day variation, which may lead to non-differential misclassification and attenuate the true associations. To partially mitigate this limitation, we used a standardized recall protocol, excluded implausible energy intakes, and adjusted for total energy intake and other key lifestyle factors in the models. In addition, we obtained similar results in sensitivity analyses restricted to participants who completed the dietary questionnaire ≥2 times, suggesting that our main findings are robust; nevertheless, residual measurement error cannot be fully ruled out. Secondly, since the cohort was based on the UK population, the association found in this study may not be generalized to other populations and should be validated in different settings and regions. Third, as the UK Biobank is an observational cohort, participants with the lowest adherence to the EAT-Lancet dietary pattern served as the reference group rather than a predefined “control” population, and residual confounding or reverse causality cannot be completely ruled out despite adjusted for key covariates (race, age, BMI, TDI, smoking, and alcohol consumption). As this is an observational cohort study, our findings cannot establish a causal relationship between adherence to the EAT-Lancet diet and uterine cancer risk and should be interpreted as associations rather than causal effects. Replication in other large-scale cohorts and, ideally, randomized or quasi-experimental intervention studies is warranted. Fourth, this analysis considered only the impact of oral contraceptives on the outcomes; the use of specific hormone therapies and different types of hormones may introduce confounding factors into the correlation. Meanwhile, we lacked metabolomic and detailed genetic data, which prevented us from exploring the underlying biological pathways and from disentangling the effect of diet from other correlated metabolic and genetic factors. Future studies that combine dietary data with metabolomic and genetic information are needed to determine whether genetic susceptibility and metabolic profiles modify the association between adherence to the EAT-Lancet diet and the risk of uterine cancer.

In summary, this study shows that following the EAT-Lancet dietary pattern relates to reduced risk of uterine cancer, and results suggest a relationship that increases with greater adherence to the pattern. Following this dietary pattern, a comprehensive approach that emphasizes plant-based foods and restricted intake of red and processed meats, provides a viable and scalable strategy for primary prevention of uterine cancer.

## Data Availability

The data analyzed in this study is subject to the following licenses/restrictions: the data that support the findings of this study are derived from the UK Biobank under approved application number [540454]. Restrictions apply to the availability of these data, which were used under license for the current study, and are therefore not publicly available. To access the dataset, please submit a request to the UK Biobank via https://www.ukbiobank.ac.uk/enable-your-research/apply-for-access.
