# Supplementary material for: Association of adherence to the EAT-Lancet diet and risk of uterine cancer: a prospective cohort from the UK Biobank
Source: Front Nutr. 2026 Jun 17;13:1801964. doi: 10.3389/fnut.2026.1801964 (PMC13318660; doi:10.3389/fnut.2026.1801964)
Supplement: Supplementary file 1 [file Table_1.docx]

**Supplementary materials**

**Association of adherence to the EAT-Lancet diet with risk of uterine cancer: a prospective cohort from the UK Biobank**

**Authors:** Lu Liu^†^, Lu Ding^†^, Zhen Chen, Lin Tang, Tianyuan Su, Nan Yang, Qi Yan, Zuoling Yang, Zhiyi Wei, Rong Li^*^, Ruxianguli Aimuzi^*^

^†^Lu Liu and Lu Ding contributed equally to this work.

^*^Ruxianguli Aimuzi and Rong Li are co-corresponding authors

**Supplemental Online Content**

**Supplementary Methods 1** Assessment of covariates.

**Supplementary Methods 2** Assessment for the Knuppel EAT-Lancet diet index.

**Table S1.** Definition of portion size and food items used in this study.

**Table S2.** Criteria for the EAT-Lancet diet index construction.

**Table S3.** Proportion of 114,572 participants adhering to the Knuppel EAT-Lancet diet index recommendations.

**Table S4.** Code used to identify uterine cancers in UK biobanking study.

**Table S5.** The breakdown of the missing data for the lifestyle factors and other covariates.

**Table S6.** Data fields and information on variables in the UK Biobank cohort involved in this study.

**Table S7.** Associations of individual food components of the Knuppel EAT-Lancet index with risks of uterine cancer.

**Table S8.** Subgroup analyses of associations between the Knuppel EAT-Lancet diet index and risks of uterine cancer.

**Table S9.** Mediating effects of BMI and Waist circumference in the associations between the EAT-Lancet indexes and risks of uterine cancer.

**Table S10-11.** Sensitivity Analysis of the effect of the Knuppel EAT-Lancet diet index on the risk of uterine cancer

**Figure S1.** Kaplan-Meier curves for uterine cancer stratified by the Knuppel EAT-Lancet diet index.

**Supplementary Methods 1**

**Assessment of covariates**

Covariates (except total energy intake) were collected at baseline (2006-2010). Covariates were made up of sociodemographic characteristics, lifestyle factors and other potential confounding factors. Sociodemographic characteristics included age (continuous), sex (males and females), ethnicity (classified as White and others), and the Townsend deprivation index (TDI). The TDI derived from the postcode of residence, was used to describe the area-based socioeconomic status through the quartiles of indices, The TDI has been validated for use in a UK-based population ^[1]^. Lifestyle covariates including smoking status (classified as never, former, current smoking and missing) and frequency of alcohol status. Physical activity, the volume of which was calculated as the sum of walking, moderate and vigorous activity over the previous week, measured as hours of physical activity, was assessed by the International Physical Activity Questionnaire (IPAQ) short form ^[2]^. Regular physical activity was defined as meeting the current global health recommendations for physical activity (150 minutes of moderate activity or 75 minutes of vigorous activity or an equivalent combination ^[3,4]^. Body mass index (BMI, kg/m^2^) was calculated as weight (kg) divided by height squared (m^2^). BMI was categorized according to the World Health Organization criteria as <18.5, 18.5-24.9, 25.0–29.9, and ≥30.0 kg/m^2^. According to the WHO classification, underweight was defined as BMI <18.5 kg/m^2^. Because only 322 women were underweight and only one uterine cancer case occurred in this group, we combined BMI <18.5 and 18.5–24.9 kg/m^2^ into a single category (<25.0 kg/m^2^) for subgroup analyses to ensure model stability. Waist circumference (cm) was measured the circumference around the level of the umbilicus at the end of the expiration period.

Total energy intake was assessed by the 24-hour dietary recall questionnaire. If participants completed dietary assessments multiple times, an average total energy intake was calculated. The details of these assessments can be found on the UK Biobank website ([www.ukbiobank](http://www.ukbiobank) ac.uk).

**Supplementary Methods 2**

**Assessment for the Knuppel EAT-Lancet diet index**

Knuppel et al. developed a quantitative index to assess adherence to the EAT-Lancet dietary recommendations. This EAT-Lancet score comprises fourteen dietary components, including whole grains, tubers and starchy vegetables, vegetables, fruit, dairy products, various protein sources, added fats, and added sugars. Each component is scored as either 0 or 1 point, depending on whether the participant's intake meets the recommended criteria, resulting in a total possible score ranging from 0 to 14, with higher scores indicating better adherence ^[5]^. Dietary intake was assessed using questionnaire data from UKB participants. Because the online 24-hour dietary assessment tool did not capture the exact weight of each food consumed, the number of standardized portions was estimated using the British Standard Food Composition Database ^[6,7]^. The consumed quantity for each food was then calculated by multiplying the number of portions by predetermined portion sizes ^[8]^. For each dietary component, a score was assigned based on whether the intake was within the EAT-Lancet recommended range (with a score of 1 for compliance, and 0 otherwise). The scores across all components were summed to produce a total EAT-Lancet index, reflecting the overall degree to which an individual’s diet aligns with the Planetary Health Diet recommendations.

Table S1. Definition of portion size and food items used in this study

| Component | Component | Food items based on the Oxford WebQ questionnaire | Code |
| --- | --- | --- | --- |
| **Whole grains** |  |  |  |
| 1 Rice, wheat, corn, and other | 1 portion: 130 g | porridge, muesli, oat crunch, sweetened cereal, plain cereal, bran cereal, whole-wheat cereal, other cereal, white pasta, wholemeal pasta, white rice, brown rice, sushi, snackpot, couscous, other grain | 100770; 100800; 100810; 100820; 100830; 100840; 100850; 100860; 102710; 102730; 102740; 102750; 102760; 102770; 102780 |
| **Tubers and starchy vegetables** |  |  |  |
| 2 Potatoes | 1 portion: 58 g | fried, boiled / baked potatoes, mashed potatoes, sweet potato | 104020; 104030; 104050; 104330 |
| **Vegetables** |  |  |  |
| 3 All vegetables | 1 portion: 100 g | quorn, mixed vegetable, vegetable pieces, coleslaw, side salad, avocado, broad bean, green bean, beetroot, broccoli, butternut squash, cabbage, carrot, cauliflower, celery, courgette, cucumber, garlic, leek, lettuce, mushroom, onion, parsnip, pea, sweet pepper, spinach, fresh tomato, tinned tomato, turnip / swede, watercress, other vegetable, sweetcorn | 103280; 104060; 104070; 104080; 104090; 104100; 104110; 104120; 104130; 104140; 104150; 104160; 104170; 104180; 104190; 104200; 104210; 104220; 104230; 104240; 104250; 104260; 104270; 104280; 104290; 104300; 104340; 104350; 104360; 104370; 104380; 104320 |
| **Fruits** |  |  |  |
| 4 All fruits | 1 portion: 150 g | stewed fruit, prune (1 portion: 48 g), dried fruit (1  portion: 48 g), mixed fruit, apple, banana, berry (1 portion: 48 g), cherry (1 portion: 48 g), grapefruit, grape (1 portion: 48 g), mango, melon, orange, satsuma, peach, pear, pineapple, plum (1 portion: 48 g), other fruit | 104410; 104420; 104430; 104440; 104460; 104470; 104480; 104490; 104500; 104510; 104530; 104540; 104550; 104560; 104570; 104580; 104590 |
| **Dairy foods** |  |  |  |
| 5 Whole milk or derivative equivalents | 1 portion: 258 g | milk, flavoured milk, yogurt (1 portion: 180 g), low fat hard cheese, hard cheese, soft cheese, blue cheese, low fat cheese spread, cheese spread, cottage cheese, feta, mozzarella, goat’s cheese, other cheese (1 portion: 28 g) | 100520; 100530; 102090; 102810; 102820; 102830; 102840; 102850; 102860; 102870; 102880; 102890; 102900; 102910 |
| **Protein sources** |  |  |  |
| 6 Beef, lamb, pork |  | bacon, ham (1 portion: 40 g), liver, sausage, beef, pork, lamb (1 portion: 80 g) | 103070; 103080; 103090; 103010; 103020; 103030; 103040 |
| 7 Chicken, other poultry | 1 portion: 80 g | crumbed or deep-fried poultry, poultry | 103050; 103060 |
| 8 Eggs | 1 portion: 60 g | whole egg, omelette, eggs in sandwiches, scotch egg, other egg | 102940; 102950; 102960; 102970; 102980 |
| 9 Fish | 1 portion: 100 g | shellfish, tinned tuna, oily fish, breaded fish, battered fish, white fish, other fish | 103220; 103150; 103160; 103170; 103180; 103190; 103230 |
| *Legumes* |  |  |  |
| 10 Dry beans, lentils, peas | 1 portion: 100 g | dried lentils, peas and baked beans | 104010 |
| 11 Soy foods | 1 portion: 100 g | tofu | 103270 |
| 12 Peanuts or tree nuts | 1 portion: 28 g | unsalted peanuts, salted nuts, unsalted nuts, seeds | 102420; 102430; 102440; 102450 |
| **Added fats** |  |  |  |
| 13 Palm oil, unsaturated oils, dairy fats (incl. in milk), lard or tallow |  | ratio of 0.8 for unsaturated: saturated fat intake^*^ | 26032; 23446; 26014 |
| **Added sugars** |  |  |  |
| 14 All sweeteners |  | Added sugars and preserves, Chocolate confectionery, Milk-dairy desserts, Oat cereal (sugar), Other cereal (sugar), Other desserts and cakes and pastries, Other sweets, Soy desserts and yogurt, Sugar-sweetened beverages and other sugary drinks | 26064; 26080; 26084; 26078; 26079; 26085; 26140; 26086; 26127; |

^*^Too little information about the types of oils were recorded in the 24-hour diet recall so fat intake was approximated as a ratio of total unsaturated and saturated fat in line with the EAT-Lancet reference diet [Reference 8]. This score is based on the work by Lu X et al.2022. [Reference 9].

Table S2. Criteria for the EAT-Lancet diet index construction

| Food Components | Scoring 0 point (g/day) | Scoring 1 point (g/day) |
| --- | --- | --- |
| **Whole grains** |  |  |
| 1 Rice, wheat, corn, and other | >464 g/day | ≤464 g/day |
| **Tubers and starchy vegetables** |  |  |
| 2 Potatoes | >100 g/day | ≤100 g/day |
| **Vegetables** |  |  |
| 3 All vegetables | <200 g/day | ≥200 g/day |
| **Fruits** |  |  |
| 4 All fruits | <1000 g/day | ≥100 g/day |
| **Dairy foods** |  |  |
| 5 whole milk or derivative equivalents | >500 g/day | ≤500 g/day |
| **Protein sources** |  |  |
| 6 Beef, lamb, and pork | >28 g/day | ≤28 g/day |
| 7 Poultry | >58 g/day | ≤58 g/day |
| 8 Eggs | >25 g/day | ≤25 g/day |
| 9 Fish and seafood | >100 g/day | ≤100 g/day |
| *Legumes* |  |  |
| 10 Dry beans, lentils, and pears | >100 g/day | ≤100 g/day |
| 11 Soy foods | >50 g/day | ≤50 g/day |
| 12 Nuts / peanuts | <25 g/day | ≥25 g/day |
| **Added fats** |  |  |
| 13 unsaturated: saturated intake | <0.8 | ≥0.8 |
| **Added sugars** |  |  |
| 14 All sweeteners | >31 g/day | ≤31 g/day |

For Added fats, the ratio of 0.8 for unsaturated: saturated fat intake received 1 point, otherwise, zero points are awarded.

The score is based on the work by Knuppel et al. 2019. [Reference 10].

Table S3. Proportion of participants adhering to the Knuppel EAT-Lancet diet index recommendations (N=114,572)

| Component | Recommendation met n (%) |
| --- | --- |
| **Whole grains** |  |
| 1 Rice, wheat, corn, and other | 114,518 (99.95) |
| **Tubers and starchy vegetables** |  |
| 2 Potatoes | 110,100 (96.10) |
| **Vegetables** |  |
| 3 All vegetables | 73,658 (64.29) |
| **Fruits** |  |
| 4 All fruits | 92,736 (80.94) |
| **Dairy foods** |  |
| 5 Whole milk or derivative equivalents | 108,220 (94.46) |
| **Protein sources** |  |
| 6 Beef, lamb, pork | 47,896 (41.80) |
| 7 Chicken, other poultry | 85,238 (74.40) |
| 8 Eggs | 84,928 (74.13) |
| 9 Fish | 111,478 (97.30) |
| *Legumes* |  |
| 10 Dry beans, lentils, peas | 110,044 (96.05) |
| 11 Soy foods | 114,088 (99.58) |
| 12 Peanuts or tree nuts | 6,913 (6.03) |
| **Added fats** |  |
| 13 Palm oil, unsaturated oils, dairy fats (incl.  in milk), lard or tallow | 112,135 (97.87) |
| **Added sugars** |  |
| 14 All sweeteners | 24,076 (21.01) |

Table S4. Code used to identify uterine cancers in UK biobanking study

| **Definition** | **Data field** | **Code** |
| --- | --- | --- |
| Uterine cancer | ICD 10 codes for: 40006 National cancer registry | C54.0; C54.1; C54.2; C54.3; C54.8; C54.9; C55 |
| Date of cancer diagnosis (national cancer registry) | 40005 |  |
| Prevalent cancer within the UK Biobank cohort was identified through national cancer registries (data fields: 40006, 40013) | | |

Abbreviations: ICD, International Classification of Diseases.

Table S5. The breakdown of the missing data for the lifestyle factors and other covariates

| Characteristic | Missing information (%) |
| --- | --- |
| Covariates | 580 (0.51) |
| Age | 0 (0.0) |
| Sex | 0 (0.0) |
| Ethnicity | 285 (0.24) |
| TDI | 295 (0.26) |
| Lifestyle factors | 5,773 (5.04) |
| Waist circumference | 181 (0.16) |
| Smoking status | 289 (0.25) |
| Alcohol status | 116 (0.10) |
| BMI | 289 (0.25) |
| Physical activity | 4,898 (4.28) |

Abbreviations: BMI, body mass index; TDI, Townsend Deprivation index

Table S6. Data fields and information on variables in the UK Biobank cohort involved in this study

| **Category** | **Data field** | **Description** |
| --- | --- | --- |
| **Demographic factors** |  |  |
| Age | 21022 | Age at recruitment |
| Sex | 31 | Sex |
| Ethnic background | 21000 | Ethnic background |
| TDI | 22189 | Townsend Deprivation index at recruitment |
| Location | 54 | UK Biobank assessment centre |
| **Lifestyle factors** |  |  |
| BMI (kg/m^2^) | 21001 | Body mass index (BMI) |
| Waist circumference (cm) | 48 | Waist circumference |
| Smoking | 20116 | Smoking status |
| Alcohol | 20117 | Alcohol drinker status |
| Physical activity | 884 | Number of days/week of moderate physical activity 10+ minutes |
|  | 904 | Number of days/week of vigorous physical activity 10+ minutes |
|  | 864 | Number of days/week of walked 10+ minutes |
|  | 894 | Duration of moderate activity |
|  | 914 | Duration of vigorous activity |
|  | 874 | Duration of walks activity |

Abbreviations: TDI, Townsend Deprivation index

Table S7. Associations of individual food components of the Knuppel EAT-Lancet index with risks of uterine cancer

| Components | EAT-Lancet index component points | | | |
| --- | --- | --- | --- | --- |
|  | 0 |  | 1 | |
|  |  |  | HR (95%CI) | *P*-value |
| Whole grains ^*^ | REF |  | - | - |
| Potatoes | REF |  | 0.914 (0.634-1.316) | 0.628 |
| Vegetables | REF |  | 0.875 (0.748-1.024) | 0.096 |
| Fruits | REF |  | 1.004 (0.820-1.229) | 0.969 |
| Dairy | REF |  | 0.843 (0.623-1.141) | 0.269 |
| Beef, lamb, and pork | REF |  | 0.887 (0.760-1.035) | 0.129 |
| Poultry | REF |  | 0.860 (0.728-1.015) | 0.075 |
| Eggs | REF |  | 1.156 (0.969-1.379) | 0.107 |
| Fish | REF |  | 1.270 (0.761-2.119) | 0.361 |
| Dry beans, lentils, peas | REF |  | 1.145 (0.761-1.722) | 0.517 |
| Soy foods | REF |  | 0.548 (0.227-1.323) | 0.181 |
| Nuts | REF |  | 0.733 (0.512-1.048) | 0.088 |
| Unsaturated oils | REF |  | 1.186 (0.685-2.055) | 0.542 |
| Added sugar | REF |  | 0.852 (0.699-1.039) | 0.113 |

^*^: In the absence of a uterine cancer outcome, Whole grains was assigned a value of 0 in 54 individuals, whereas in the presence of a uterine cancer outcome, Whole grains was assigned a value of 0 in 0 individuals, and therefore HRs, 95% CIs and p-values were not calculated for this outcome.

Models were adjusted for age, Townsend scores, ethnicity, smoking status, alcohol intake, physical activity and energy intake.

Table S8. Subgroup analyses of associations between the Knuppel EAT-Lancet diet index and risks of uterine cancer

|  | EAT-Lancet diet index categories | | | | | | | | | *P* for trend | 1-point increment in diet score | *P* for interaction |
| --- | --- | --- | --- | --- | --- | --- | --- | --- | --- | --- | --- | --- |
|  | ≤9 | =10 | |  | =11 | |  | ≥12 | |  |  |  |
|  |  | HR (95%CI) | *P*-value |  | HR (95%CI) | *P*-value |  | HR (95%CI) | *P*-value |  |  |  |
| **Age** |  |  |  |  |  |  |  |  |  |  |  | 0.811 |
| <56 | REF | 1.075 (0.758-1.525) | 0.686 |  | 1.043 (0.730-1.492) | 0.816 |  | 0.657 (0.410-1.052) | 0.080 | 0.157 | 0.956 (0.859-1.035) |  |
| ≥56 | REF | 0.946 (0.730-1.224) | 0.672 |  | 0.960 (0.743-1.239) | 0.752 |  | 0.737 (0.543-1.001) | 0.050 | 0.087 | 0.926 (0.857-1.000) |  |
| **Townsend scores** |  |  |  |  |  |  |  |  |  |  |  | 0.563 |
| Above median | REF | 1.090 (0.671-1.198) | 0.459 |  | 0.948 (0.713-1.261) | 0.715 |  | 0.750 (0.534-1.053) | 0.097 | 0.044 | 0.956 (0.878-1.042) |  |
| Below median | REF | 1.068 (0.791-1.441) | 0.668 |  | 0.990 (0.731-1.342) | 0.948 |  | 0.649 (0.441-0.956) | 0.029 | 0.236 | 0.900 (0.821-0.987) |  |
| **Smoking status** |  |  |  |  |  |  |  |  |  |  |  | 0.340 |
| Never | REF | 0.979 (0.752-1.274) | 0.872 |  | 1.095 (0.847-1.417) | 0.487 |  | 0.686 (0.495-0.951) | 0.024 | 0.162 | 0.945 (0.874-1.023) |  |
| Previous / current | REF | 0.983 (0.701-1.378) | 0.919 |  | 0.761  (0.531-1.089) | 0.126 |  | 0.750 (0.498-1.127) | 0.166 | 0.078 | 0.909 (0.819-1.009) |  |
| **Alcohol intake** |  |  |  |  |  |  |  |  |  |  |  | 0.351 |
| Never | REF | 1.103 (0.549-2.215) | 0.782 |  | 0.425 (0.180-1.001) | 0.050 |  | 0.459 (0.170-1.236) | 0.123 | 0.018 | 0.789 (0.623-1.000) |  |
| Previous / current | REF | 0.966 (0.777-1.201) | 0.757 |  | 1.019 (0.822-1.264) | 0.862 |  | 0.729 (0.559-0.949) | 0.019 | 0.093 | 0.942 (0.882-1.005) |  |
| **Physical activity** |  |  |  |  |  |  |  |  |  |  |  | 0.390 |
| irregular | REF | 1.140 (0.845-1.539) | 0.392 |  | 0.966 (0.705-1.323) | 0.828 |  | 0.724 (0.487-1.075) | 0.100 | 0.107 | 0.936 (0.852-1.028) |  |
| regular | REF | 0.843 (0.632-1.125) | 0.247 |  | 0.957 (0.725-1.263) | 0.758 |  | 0.682 (0.488-0.953) | 0.025 | 0.118 | 0.926 (0.851-1.008) |  |
| **BMI** |  |  |  |  |  |  |  |  |  |  |  |  |
| <25.0 | REF | 1.392  (0.882-2.196) | 0.155 |  | 1.480  (0.947-2.313) | 0.085 |  | 1.012  (0.606-1.690) | 0.963 | 0.956 | 1.002  (0.889-1.129) | 0.830 |
| 25.0-29.9 | REF | 0.968  (0.693-1.371) | 0.823 |  | 0.937  (0.659-1.333) | 0.717 |  | 0.865  (0.571-1.310) | 0.494 | 0.488 | 0.973  (0.875-1.082) |  |
| ≥30 | REF | 1.027  (0.747-1.412) | 0.869 |  | 1.155  (0.836-1.595) | 0.382 |  | 0.852  (0.549-1.322) | 0.475 | 0.941 | 1.006  (0.908-1.115) |  |

Models were adjusted for age, Townsend scores, ethnicity, smoking status, alcohol intake, physical activity and energy intake.

BMI categories were defined according to WHO criteria, with underweight (<18.5 kg/m^2^) combined with normal weight (18.5–24.9 kg/m^2^) due to the small number of cases (1 uterine cancer case among 322 underweight participants).

Table S9. Mediating effects of BMI and Waist circumference in the associations between the EAT-Lancet indexes and risks of uterine cancer

| Uterine cancer | BMI | |  | Waist circumference | |
| --- | --- | --- | --- | --- | --- |
|  | HR (95%CI) | Mediation Proportion (95% CI) |  | HR (95%CI) | Mediation Proportion (95% CI) |
| Direct Effect | 0.852 (0.826-0.881) |  |  | 0.837 (0.645-1.082) |  |
| Indirect Effect | 0.851 (0.651-1.100) |  |  | 0.855 (0.824-0.887) |  |
| Mediation proportion |  | 0.494 (0.249-1.908) ^a^ |  |  | 0.743 (0.243-1.905) ^b^ |

^a^:*P*-value=0.014; ^b^: *P*-value=0.006

Models were adjusted for age, Townsend scores, ethnicity, smoking status, alcohol intake, physical activity and energy intake.

Table S10. Sensitivity Analysis of the effect of the Knuppel EAT-Lancet diet index on the risk of uterine cancer

| Knuppel EAT-Lancet index | Model 1 | Model 2 | Model 3 | Model 4 |
| --- | --- | --- | --- | --- |
| ≤9 | REF | REF | REF | REF |
| 10 | 0.800 (0.620-1.033) | 0.977 (0.794-1.203) | 0.961 (0.775-1.190) | 0.985 (0.760-1.276) |
| 11 | 0.898 (0.698-1.156) | 0.969 (0.787-1.193) | 0.952 (0.768-1.180) | 0.975 (0.752-1.264) |
| ≥12 | 0.737 (0.538-1.009) | 0.707 (0.548-0.913) | 0.706 (0.544-0.918) | 0.764 (0.560-1.043) |
| *P* for trend | 0.144 | 0.017 | 0.020 | 0.128 |
| 1-point increment in diet score | 0.953 (0.880-1.033) | 0.931 (0.875-0.992) | 0.931 (0.873-0.994) | 0.951 (0.880-1.028) |

Model 1: Excluding participants with only one dietary recall questionnaire (N=70,154).

Model 2: Follow-up time which began at the time of the completion of the latest dietary assessment (N=114,572).

Model 3: Excluding participants with covariate-filled data (N=108,927).

Model 4: Excluding uterine cancer cases that occurred within the first five years of follow-up (N=114,329).

Table S11. Sensitivity Analysis of the effect of the Knuppel EAT-Lancet diet index on the risk of uterine cancer

| Knuppel EAT-Lancet index | Model 1 | Model 2 |
| --- | --- | --- |
| ≤9 | REF | REF |
| 10 | 1.017 (0.794-1.302) | 0.923 (0.731-1.166) |
| 11 | 1.066 (0.835-1.362) | 0.889 (0.702-1.125) |
| ≥12 | 0.730 (0.539-0.987) | 0.642 (0.479-0.860) |
| *P* for trend | 0.148 | 0.004 |
| 1-point increment in diet score | 0.947 (0.880-1.019) | 0.901 (0.837-0.968) |

Model 1: Excluding participants with a parental family history of cancer (N=85,656).

Model 2: Excluding participants with used oral contraceptive pill during the study period (N=96,821).


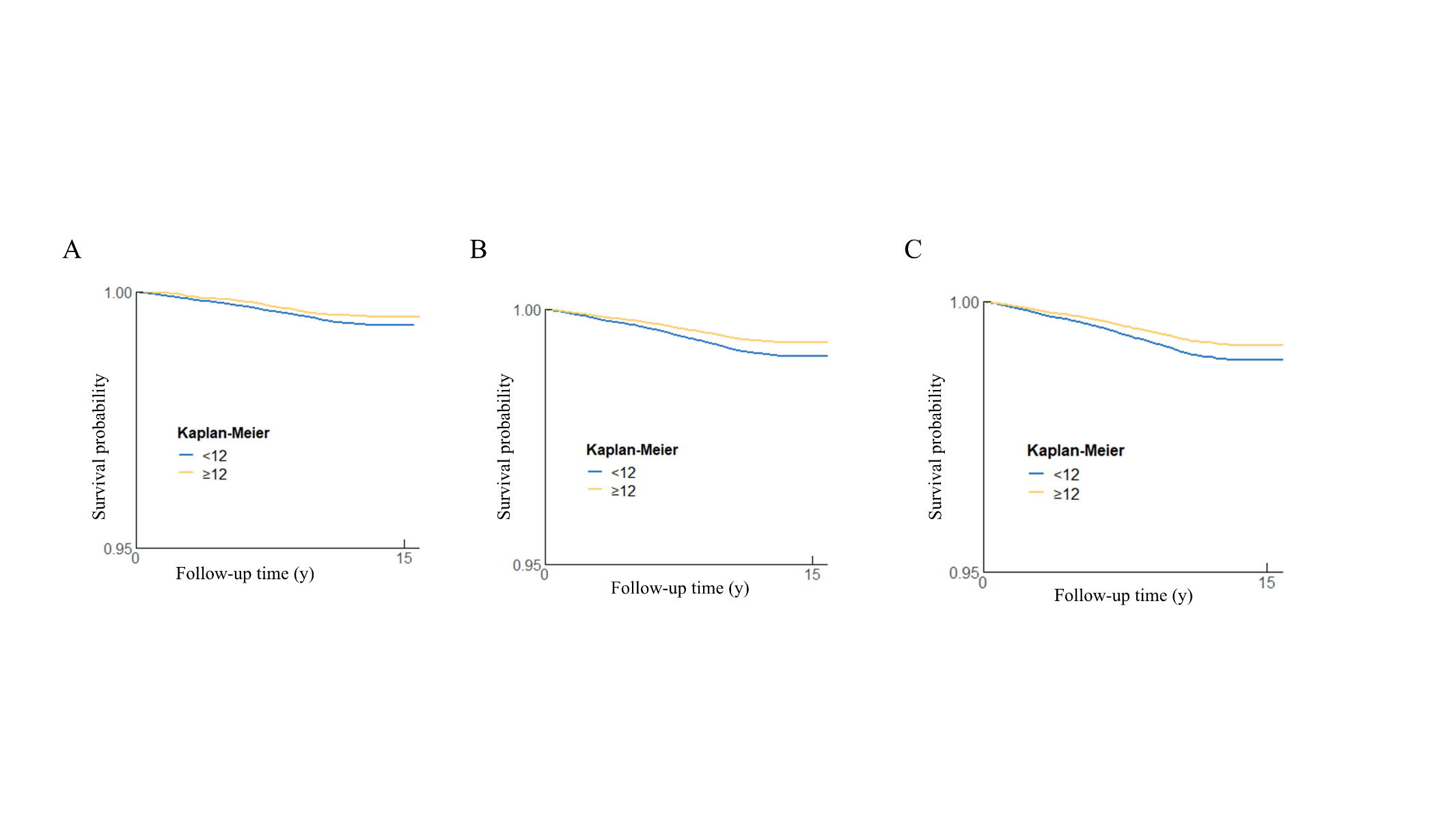


**Figure S1**. Kaplan-Meier curves for uterine cancer stratified by the Knuppel EAT-Lancet diet index.

Unadjusted (A), with adjusted for age, Townsend scores and ethnicity (B), with adjusted for age, Townsend scores, ethnicity, smoking status, alcohol intake, physical activity and energy intake (C).

**References**

1. Townsend P: Poverty in the United Kingdom In London: Allen Lane and Penguin Books; 1979.
2. Craig CL, Marshall AL, Sjostrom M, Bauman AE, Booth ML, Ainsworth BE, et al. International physical activity questionnaire: 12-country reliability and validity. Medicine and science in sports and exercise 2003;35(8):1381-95.
3. NHS. Physical activity guidelines for adults 2018.
4. Physical Activity Guidelines Advisory Committee. 2018 Physical Activity Guidelines Advisory Committee Scientific Report U.S2018.
5. Knuppel A, Papier K, Key TJ, Travis RC. EAT-Lancet score and major health outcomes: the EPIC-Oxford study. Lancet 394, 213-214 (2019).
6. Ministry of Agriculture Fisheries and Food. Food Portion Sizes. 2nd ed. London: HMSO (1993).
7. Public Health England. Composition of Foods Integrated Dataset (CoFID). (2019). Available online at: https://www.gov.uk/government/publications/composition-of-foods-integrated-dataset-cofid (accessed January 22, 2021).
8. Xu C, Cao Z, Yang H, Hou Y, Wang X, Wang Y. Association Between the EAT-Lancet Diet Pattern and Risk of Type 2 Diabetes: A Prospective Cohort Study. Front Nutr 8, 784018 (2022).
9. Lu X, Wu L, Shao L, Fan Y, Pei Y, Lu X, Borné Y, Ke C. Adherence to the EAT-Lancet diet and incident depression and anxiety. Nat Commun. 2024 Jul 3;15(1):5599.
10. Knuppel A, Papier K, Key TJ, Travis RC. EAT-Lancet score and major health outcomes: the EPIC-Oxford study. Lancet. 2019; 394: 213-214.
